# A review of candidate therapies for Middle East respiratory syndrome from a molecular perspective

**DOI:** 10.1099/jmm.0.000565

**Published:** 2017-08-31

**Authors:** Ali A. Rabaan, Shamsah H. Alahmed, Ali M. Bazzi, Hatem M. Alhani

**Affiliations:** ^1^​ Molecular Diagnostic Laboratory, Johns Hopkins Aramco Healthcare, Dhahran 31311, Saudi Arabia; ^2^​ Specialty Paediatric Medicine, Qatif Central Hospital, Qatif 32654, Saudi Arabia; ^3^​ Microbiology Laboratory, Johns Hopkins Aramco Healthcare, Dhahran 31311, Saudi Arabia; ^4^​ Maternity and Children Hospital, and Directorate of Infection Control at Eastern Province, Ministry of Health, Dammam, Saudi Arabia

**Keywords:** Antiviral peptide, camostat, convalescent plasma, DPP4, GLS-5300, glycopeptide antibiotic, interferon, lopinavir, MERS-CoV, monoclonal antibodies, M(pro), PL(pro), protease, S protein, vaccine

## Abstract

There have been 2040 laboratory-confirmed cases of Middle East respiratory syndrome coronavirus (MERS-CoV) in 27 countries, with a mortality rate of 34.9 %. There is no specific therapy. The current therapies have mainly been adapted from severe acute respiratory syndrome (SARS-CoV) treatments, including broad-spectrum antibiotics, corticosteroids, interferons, ribavirin, lopinavir–ritonavir or mycophenolate mofetil, and have not been subject to well-organized clinical trials. The development of specific therapies and vaccines is therefore urgently required. We examine existing and potential therapies and vaccines from a molecular perspective. These include viral S protein targeting; inhibitors of host proteases, including TMPRSS2, cathepsin L and furin protease, and of viral M(pro) and the PL(pro) proteases; convalescent plasma; and vaccine candidates. The Medline database was searched using combinations and variations of terms, including ‘Middle East respiratory syndrome coronavirus’, ‘MERS-CoV’, ‘SARS’, ‘therapy’, ‘molecular’, ‘vaccine’, ‘prophylactic’, ‘S protein’, ‘DPP4’, ‘heptad repeat’, ‘protease’, ‘inhibitor’, ‘anti-viral’, ‘broad-spectrum’, ‘interferon’, ‘convalescent plasma’, ‘lopinavir ritonavir’, ‘antibodies’, ‘antiviral peptides’ and ‘live attenuated viruses’. There are many options for the development of MERS-CoV-specific therapies. Currently, MERS-CoV is not considered to have pandemic potential. However, the high mortality rate and potential for mutations that could increase transmissibility give urgency to the search for direct, effective therapies. Well-designed and controlled clinical trials are needed, both for existing therapies and for prospective direct therapies.

## Introduction

### Middle East respiratory syndrome coronavirus overview

Middle East respiratory syndrome coronavirus (MERS-CoV) was first isolated in Jeddah in the Kingdom of Saudi Arabia (KSA) from a 60-year-old male hospital patient, who died 24 June 2012, 11 days after presenting with acute pneumonia and subsequent renal failure [1]. Since then, the WHO have been notified of 2040 laboratory-confirmed cases, including 712 deaths [2]. While most cases have arisen in the Middle East, cases have also emerged in 27 countries worldwide in travellers from the Middle East and/or in their contacts [2].

Most human MERS-CoV infections are considered to be the result of multiple zoonotic transfers. Bats are the most likely MERS-CoV natural reservoir, as with other mammalian coronaviruses (CoVs), while camels are likely to be the major zoonotic source for human infections [3–5]. Secondary human-to-human transmission is considered to be limited, occurring mainly within family and healthcare settings. The first cluster of cases in humans was retrospectively identified to have occurred in a public hospital in Jordan in April 2012 [6]. Multiple healthcare facility-associated outbreaks have since occurred in the Middle East, most notably in KSA, often linked to deficiencies in infection control procedures [7–13]. Although cases outside the Middle East have mainly been isolated, a large outbreak occurred in Korea in June 2015, in which human–human transmission resulted in 186 cases and 36 deaths [14]. Increased vulnerability to either cross-species or trans-human transmission could result from viral adaptations [15].

MERS-CoV infection is often accompanied by acute viral pneumonia, and sometimes gastrointestinal symptoms. Clinical severity varies from asymptomatic to death, and the extent of asymptomatic spread is unclear. The high mortality rate is mainly accounted for by acute respiratory distress syndrome (ARDS) [7, 15, 16]. Higher mortality is observed among vulnerable patients, such as older individuals and those suffering from comorbid illness, and is also associated with high viral load [7, 11, 15, 17]. In one study in a KSA hospital, intensive care unit (ICU) admission among MERS-CoV patients was associated with a mortality rate of 74.2 % [11].

While MERS-CoV is not currently considered to have pandemic potential, it is clear that human–human transmission does occur. The exact mechanisms by which MERS-CoV is transmitted from animals to humans have not been fully elucidated. In the South Korean outbreak, the virus emerged in second- and third-generation contacts, resulting in the first human case to be imported into China [18]. This raised concern that viral mutations were contributing to human–human transmission.

Given its high mortality and poor outcomes for vulnerable patients, and the potential for viral mutations, there is no room for complacency in the search for therapeutic options for MERS-CoV. There is currently no specific therapy. Many of the therapeutic options used have been adapted from approaches used to treat severe acute respiratory syndrome (SARS-CoV) during the outbreak of 2003, and/or the H1N1 influenza virus during the outbreak of 2009 [19]. However, while MERS-CoV and SARS-CoV are phylogenetically related betacoronaviruses, they differ in many important respects. MERS-CoV utilizes human dipeptidyl peptidase 4 (DPP4; CD26) receptors, with binding mediated by the viral spike (S) protein, not the angiotensin-converting enzyme 2 (ACE-2) receptors used by SARS [20–24]. MERS-CoV also has a wider cellular tropism [24–26].

Therapies currently used include broad-spectrum antibiotics, corticosteroids and anti-viral treatments, such as interferons (IFN), ribavirin, lopinavir–ritonavir, or mycophenolate mofetil [19, 22, 27–31]. However, the efficacy and/or safety of many of these therapies is unclear, and none are specific to MERS-CoV. Ribavirin monotherapy, for example, is associated with multiple side-effects in the treatment of other viral illnesses, including SARS-CoV, has uncertain efficacy, and has not been tested in animal studies or randomized control trials for MERS-CoV [22, 31, 32]. Corticosteroids have not been successful in the treatment of respiratory distress or lung fibrosis in MERS-CoV [31, 32]. Meanwhile, studies in SARS-CoV and H1N1 patients suggest that corticosteroid use may in fact increase viral replication in airways, and SARS patient and animal studies indicate that it contributes to immunosuppression [33–35]. Mycophenolate mofetil has been associated with fatal disease and high viral loads in a marmoset model of MERS-CoV infection [36]. IFN therapy, alone or in combination with ribavirin or lopinavir–ritonavir, has shown greater promise in *in vitro*, animal and human studies [37–40]. However, clinical studies on IFNs vary with respect to factors such as time of administration and type of patient [19, 22, 40]. Overall, there is a lack of randomized control trials (RCTs) designed to test the safety and efficacy of any potential therapies specific to MERS-CoV, and much of the information available for existing therapies is based on *in vitro* and/or animal studies [22, 40, 41]. A position paper on the evidence base for specific MERS-CoV therapies, published by Public Health England (PHE) and the World Health Organization*–*International Severe Acute Respiratory and Emerging Infection Consortium (ISARIC*–*WHO), suggested that benefit was likely to exceed risk for convalescent plasma, lopinavir*–*ritonavir, IFNs and monoclonal/polyclonal antibodies, while, by contrast, for ribavirin monotherapy and corticosteroids it was considered that the risks would outweigh the benefits [42]. For interferon/ribavirin combination therapy, nitazoxanide and chloroquine, the available data were considered to be inadequate for assessment [42].

In this review, we consider potentially effective MERS-CoV therapies, including IFNs, lopinavir*–*ritonavir and inhibitors of proteases, including TMPRSS2 and cathepsin L, as well as MERS-CoV-specific potential therapies, including convalescent plasma, monoclonal antibodies (mAbs), antiviral peptides and candidate vaccines. These therapies will be considered from a molecular perspective, in the context of the infection and replication mechanisms of MERS-CoV. The therapies are summarized in Table 1.

**Table 1.** Summary of potential MERS-CoV therapies

**Target**	**Type**	Therapy/vaccine	*In vivo/in vitro*	Safety/advantages	Side-effects/disadvantages	References
S1/DPP4 binding	Antibody (mouse): S1 RBD	Mersmab	*In vitro*			[76]
	Antibody (human): S1 RBD	m336, m337, m338	*In vitro/in vivo* (mouse, rabbit – m336)			[77–79]
	Antibody (human): S1 RBD	MERS-4, MERS-27	*In vitro*			[80, 81]
	Antibody (mouse- humanized): S1 RBD	4C2	*In vitro/in vivo* (mouse)	Prophylactic and therapeutic		[82]
	Antibody (mouse- humanized): S1 RBD	hMS-1	*In vitro/in vivo* (mouse)			[83]
	Antibody (human): S1 RBD	LCA60	*In vitro/in vivo* (mouse)	Targets both NTD and RBD; stable CHO cell line; prophylactic and therapeutic		[84]
	Antibody (human): S1 RBD	3B11-N	*In vitro/in vivo* (rhesus monkeys)	Prophylactic		[85]
	Antibody (human- anti-DPP4)	2F9, 1F7, YS110	*In vitro*			[86]
	RBD-IgG fusion vaccine candidate	RBD s377-588- Fc IgG fusion	*In vitro/in vivo* (mouse)	Humoral response in mice; potential intranasal administration; improved by adjuvant MF59; divergent strains/escape mutants		[91–95]
	Full-length S protein proprietary nanoparticles		*In vitro/in vivo* (mouse)	Use of adjuvants improves humoral response	Stable expression of abundant full-length S protein difficult	[97]
	MVA expressing full-length S protein (vaccine candidate)		*In vitro/In vivo* (mouse, camel)	T cell and humoral response; entering human clinical trials; potential for veterinary use – camels		[98, 99]
	ad5 or ad41 adenovirus expressing full-length S (vaccine candidate)		*In vitro/in vivo* (mouse)	T cell and neutralizing antibody responses		[99]
	Measles virus expressing full-length S (vaccine candidate)		*In vitro/in vivo* (mouse)	T cell and neutralizing antibody responses		[100]
	Plasmid vaccine	GLS-5300	*In vitro/in vivo* (mouse, camels and macaques) Human clinical trials	T cell and neutralizing antibody responses; in phase I clinical trial		[102, 103]
Viral S2-host membrane fusion	Anti-HR2 viral peptide	HR2P	*In vitro*			[87]
	Anti-HR2 viral peptide	HR2P-M2	*In vitro/In vivo* (mouse)	Blocks 6HB bundle formation; enhances IFN-β effect; potential intranasal treatments		[88–90]
Immune evasion response	IFN-α2b and ribavirin		*In vitro/In vivo* (macaque)	Combination therapy allows reduced amounts of each; non-human primate model; 10 different gene pathways		[108–110]
	IFN-β1b and lopinavir		*In vitro/In vivo* (marmoset)	Combination therapy allows reduced amounts of each		[111]
	IFN combination therapy (ribavirin and/or lopinavir		Case studies (human)		Needs to be used prophylactically or early for any clinical benefit; insufficient evidence of clinical efficacy as yet	[37–40]
	IFN combination therapy (ribavirin)		Retrospective cohort studies (human)	Probable benefit of early use in less vulnerable patients; safety and efficacy established for other viral illnesses	Needs to be used prophylactically or early for any clinical benefit; insufficient evidence of clinical efficacy as yet	[27, 29, 105, 107, 112, 113]
S protein host proteases	TMPRSS2 inhibitor	Camostat	*In vivo* – mouse, SARS-CoV	Already in clinical use (chronic pancreatitis)		[59]
	TMPRSS2 inhibitor	Nafamostat	Split-protein-based cell–cell fusion assay	Already in clinical use (anti-coagulant)		[118]
	Cathepsin L inhibitor	Teicoplanin dalbavancin oritavancin telavancin	High-throughput screening	Already in clinical use (antibiotic Gram-positive bacterial infections)		[119]
Viral proteases	PL(pro) inhibitor	6-mercaptopurine (6MP) 6-thioguanine (6TG)	*In vitro*	Potential for more MERS-specific agents		[61]
	PL(pro) inhibitor	F2124–0890	*In vitro*		May lose potency in physiological reducing environments	123]
	Mpro	Lopinavir	*In vitro/In vivo* (marmosets)	High activity in low micromolar range *in vitro*; better outcomes, reduced mortality in marmosets	Clinical efficacy not fully established in humans	[36, 47, 125]

## MERS-CoV infection and replication

### MERS-CoV lineage and structure

**Fig. 1.**
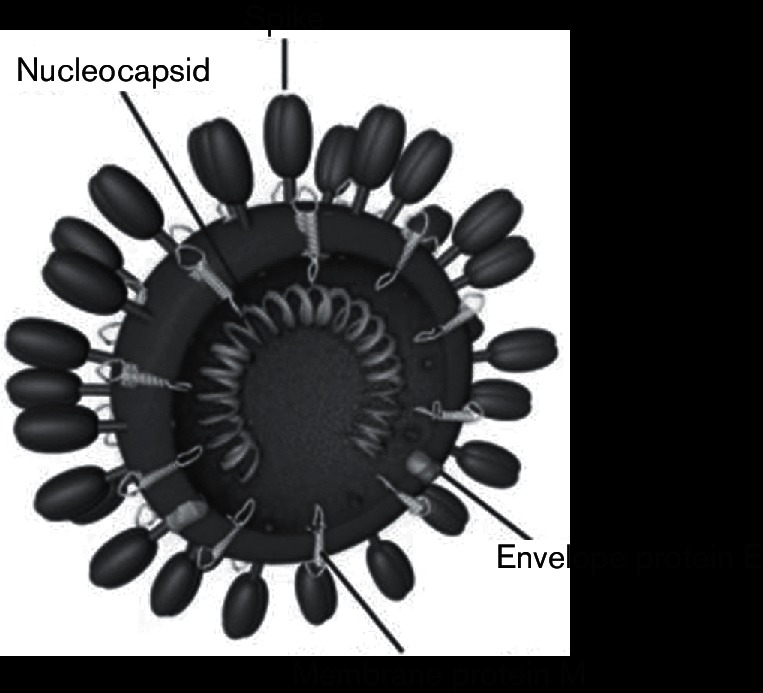
Structure of MERS-CoV. Taken from: Belouzard *et al*. [128].

**Fig. 2.**
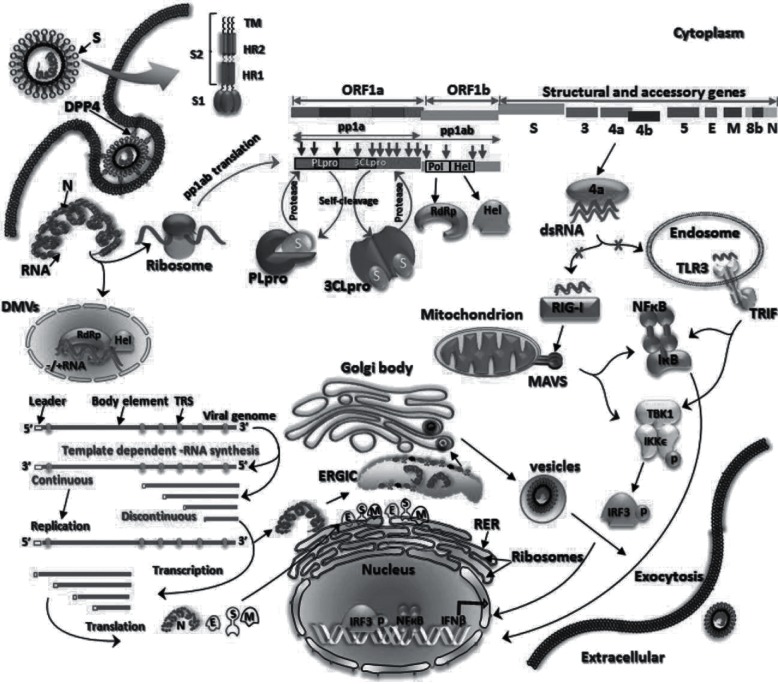
Replication cycle and potential therapeutic targets of MERS-CoV. Adapted from Durai *et al.* [57].

MERS-CoV is a betacoronavirus belonging to clade c (lineage 3) of the betacoronaviruses [43]. Its closest known coronavirus relatives are the prototypic clade c betacoronaviruses, *Tylonycteris* bat virus HKU4, *Pipistrellus* bat HKU5 virus and *Neoromicia zuluensis* bat PML/2011 (NeoCoV) virus [1, 43–47]. In common with other coronaviruses, the genome of MERS-CoV is a single, positive-stranded RNA of over 30 000 nucleotides. It encodes 10 predicted open reading frames (ORFs) and genes for 4 structural proteins, namely the spike (S), nucleocapsid (N), membrane (M) and envelope (E) proteins (Figs 1 and 2) [48–50]. ORF 1a and 1b encode virus replication-related proteins (pp1a, pp1ab), which are cleaved to give 16 non-structural proteins (NSPs) involved in synthesis of viral RNA and recombination (Fig. 2) [48–50]. These include NSP-14, which contains a 39-to-59 exoribonuclease (ExoN) domain that is important in viral proofreading and in determining the sensitivity of RNA viruses to mutagens. Thus small-molecule inhibitors of ExoN activity could be candidates for MERS-CoV and other coronavirus therapies [51]. As with other coronaviruses, the MERS-CoV S protein is critical to host cell receptor binding and cell entry, and is considered to have been under strong positive selection pressure when the virus was transmitted to humans [52, 53]. Hence the S protein is a major target for potential anti-MERS-CoV therapies [53].

### MERS-CoV Spike (S) protein

The S protein of MERS-CoV is composed of S1 and S2 subunits (Fig. 2) [53]. In common with other coronaviruses, entry into host cells depends on the S1 subunit, which contains a receptor-binding domain (RBD) comprising a core subdomain and a receptor-binding motif (RBM). The MERS-CoV RBM differs from that of SARS-CoV and dictates that MERS-CoV uses the DPP4 receptor, as opposed to the ACE-2 receptor [20, 21]. The infection process is shown in Fig. 2. DPP4, which is widely expressed in tissues, including the lung and kidneys, is critical in the species tropism of MERS-CoV infection; bat, human, camel, non-human primate and swine cells, for example, are permissive for MERS-CoV infection, whereas mouse, hamster and ferret are not [54, 55]. Host species restriction has been attributed to differences in five amino acids involved in DPP4-RBD binding, with glycosylation of the mouse DPP4 also identified as being important in the inhibition of MERS-CoV infection [54–56]. The human DPP4 receptor is therefore a potential target for MERS-CoV-specific therapeutics, in particular anti-DPP4 mAbs (Fig. 2, Table 1) [53–56]. Adenosine deaminase (ADA), which is a DPP4-binding protein, competes with MERS-CoV for DPP4 binding and hence is a natural MERS-CoV antagonist; this gives potential insights for the development of therapeutic antagonists [55].

MERS-CoV binds to the permissive host cell DPP4 via the RBD of the S1 domain, one of the major targets for potential MERS-CoV therapies [53]. Similarly to other coronaviruses, MERS-CoV then uses the S2 subunit for virus–host membrane fusion (Fig. 2). Fusion results in cleavage of the S protein at the S1/S2 boundary by host proteases [57]. The S2 subunit contains the fusion peptide, two heptad repeat domains termed HR1 and HR2, and a transmembrane (TM) domain (Fig. 2) [57]. Membrane fusion requires conformational rearrangement of S2, the formation of a six-helix bundle (6HB) fusion core, of which HR1 and HR2 are essential elements, and exposure of the fusion peptide, which inserts itself into the host cell membrane [52, 57, 58]. HR2-derived peptides have been identified as potentially effective anti-viral agents for treatment of MERS-CoV [52] (Fig. 2; Table 1).

### Host cell proteases and MERS-CoV infection

The availability of host cell proteases is essential for MERS-CoV entry into cells [23, 53]. The host proteases responsible for S protein cleavage at the S1/S2 boundary include the serine protease TMPRSS2, endosomal cathepsins such as cathepsin L, and furin protease [23,  53, 59–61]. *In vitro* studies suggest that uncleaved MERS pseudovirus can enter host cells by cathepsin l-dependent endocytosis, but that cleavage of virus during maturation by host proteases such as TMPRSS2 results in viral entry at neutral pH and the formation of massive syncytia [58]. Host cell proteases are therefore potential molecular therapeutic targets for MERS-CoV prophylaxis and/or treatment (Fig. 2, Table 1). The TMPRSS2 inhibitor camostat, for example, has been identified as a potential therapeutic agent for coronaviruses such as SARS-CoV and MERS-CoV [59].

Following host cell entry, MERS-CoV pp1a and pp1ab are synthesized and then cleaved by two viral proteases, the main protease (Mpro/3CLpro) and the papain-like protease (PLpro) (Fig. 2) [57, 63]. Thus viral proteases represent further potential molecular targets for therapy (Table 1). The recently described MERS-CoV Mpro crystal structure resembles other coronavirus Mpro proteases [60]. The SARS-CoV PL(pro) inhibitors, 6-mercaptopurine (6MP) and 6-thioguanine (6TG), can inhibit MERS CoV protease activity *in vitro*, as can the immunosuppressant drug mycophenolic acid [61]. However, caution is required, as the results of studies on marmosets have associated use of mycophenolate mofetil with fatal disease and high viral loads [36].

### MERS-CoV proteins and immune system circumvention

Other MERS-CoV proteins involved in helping the virus to circumvent the immune system also present potential molecular targets (Fig. 2). For example, the accessory protein products of ORF 4a, 4b and 5 are interferon (IFN) antagonists [62, 63]. The ORF 4a protein both inhibits type I IFN production via cytoplasmic and nuclear mechanisms, and interferes with the IFN-mediated interferon-stimulated response element (ISRE) promoter element signalling pathways [62, 63]. This gives a molecular level rationale for the use of IFN as a therapeutic option in MERS-CoV treatment. MERS-CoV can also infect dendritic cells and macrophages [26, 64, 65]. Endosomal uptake of MERS-CoV by dendritic cells following binding via DPP4 prompts these cells to produce abundant amounts of type I and III IFNs [65]. This gives context to the IFN antagonism exhibited by MERS-CoV accessory proteins. Recently, MERS-CoV has also been shown to infect T cells, which are rich in DPP4, both *in vitro* and in marmoset spleen [66]. This results in T cell apoptosis and could contribute significantly to viral pathogenesis and further emphasizes the potential therapeutic utility of molecular targeting of DPP4 and/or the MERS-CoV S protein. Both convalescent plasma containing virus-specific antibodies and the use of specific mAbs provide options for targeting MERS-CoV infection at a molecular level.

## Convalescent plasma

The use of convalescent plasma [or hyperimmune IV immunoglobulin (HVIG) from the plasma of convalescent donors] for infectious disease treatments has a long history, including in the treatment of respiratory diseases [67–70]. For influenza and SARS-CoV infection, early convalescent plasma treatment within 4–5 days of symptoms is associated with decreased viral load and reduction in mortality [67–70]. However, for SARS-CoV the quality of studies has been inconsistent and the results have been inconclusive, with a lack of adequate clinical trials [69, 70]. According to the PHE and ISARIC–WHO position paper, convalescent plasma (or high neutralizing antibody titre products) is indicated for the treatment of serious MERS-CoV infection [42]. One RCT on 35 critically ill patients with H1N1 infection identified a significant reduction in viral load and mortality in patients who received HVIG within 5 days of the onset of symptoms [68]. To date, no RCTs have been completed on the use of convalescent plasma/HVIG in MERS-CoV patients. In the light of results from SARS and influenza patients, an ongoing clinical trial on the safety and efficacy of convalescent plasma treatment for critically ill MERS-CoV patients was initiated in May 2014 and is due to report in June 2017 [75; ClinicalTrials.gov identifier: NCT02190799]. This trial is being carried out in KSA. However, as is common for convalescent plasma therapies, the trial has been affected by logistical and technical issues, including the availability of sufficient donors [71, 72]. Issues can also arise in the collection of convalescent plasma that has sufficient levels of MERS-CoV antibodies, particularly outside the Middle East [22, 72]. While there are two case reports in which intravenous immunoglobulin (IVIG) was used in treatment of MERS-CoV, it is uncertain as to whether this contributed to patient recovery [73, 74]. Thus, while convalescent plasma is a promising potential therapy for MERS-CoV, the available clinical evidence is very limited and the results of the ongoing clinical trial will be vital in guiding any future use [71]. Focused development of neutralizing monoclonal antibodies targeted against specific MERS-CoV proteins has meanwhile yielded promising *in vitro* and/or *in vivo* results.

## Monoclonal antibodies: S1-DPP4 binding

A number of mouse and human neutralizing mAbs against the S1 region of MERS-CoV have been developed and tested *in vitro* and/or in animal models [52]. Targeting of S protein for therapeutic purposes was recently comprehensively reviewed by Du *et al.* [53] [52]. In particular, the S1 RBD is a popular target, as mAbs directed against this region have the most potent neutralizing capacity. However, in terms of vaccine development, neutralizing antibodies raised by immunization with full-length S or S1 protein expression vectors may produce a more effective immunogenicity through the targeting of multiple epitopes and the reduction of the possibility of escape mutations [75]. Nevertheless, many mouse and human mAbs targeting the S1 RBD have given promising results *in vitro* and in mouse models [19]. (Table 1). A mouse monoclonal antibody, Mersmab1, blocks MERS pseudovirus cell entry *in vitro* by binding to the RBD and preventing S1 binding to DPP4 [76]. Meanwhile, the human monoclonal antibodies m336, m337 and m338, which target overlapping epitopes in the RBD, all potently neutralize pseudovirus and live MERS-CoV *in vitro* [77]. Significantly, intraperitoneal injection of m336 has also been shown to have both prophylactic and therapeutic protective effects against MERS-CoV infection in a well-established human DPP4 (hDPP4)-expressing transgenic mouse model, and in rabbits [78, 79]. Other anti-RBD human antibodies, MERS-4 and MERS-27, which recognize distinct RBD regions and block binding to DPP4, likewise have potent *in vitro* neutralizing activity against pseudovirus and live virus infection, and they also act synergistically [80, 81]. MERS-4 has anti-syncytia formation activity [80]. The crystal structure of MERS-27 bound to the DPP4 receptor revealed two critical RBD residues [81]. The crystal structure of another anti-RBD antibody 4C2, which was raised in mice, has also been elucidated. This has allowed the identification of an epitope that partially overlaps the RBD receptor binding unit [82]. 4C2 was consequently humanized to give an antibody with prophylactic and therapeutic properties, shown by a reduction of MERS-CoV lung viral titres in an Ad5-hCD26 (hDPP4) transgenic mouse model [82]. Another humanized anti-RBD antibody, hMS-1, similarly has potent *in vivo* protective properties against fatal MERS-CoV infection in a transgenic hDPP4 mouse model [83]. The human antibody LCA60 targets both the N-terminal domain (NTD) and the RBD of the S1 region, and was isolated from B cells of a MERS-CoV-infected human donor before being used to rapidly establish a stable CHO cell line that can be used to reliably produce clinical grade antibody [84]. This is a promising candidate for clinical development, given the antibody’s potent prophylactic and therapeutic activities against MERS-CoV infection in Ad5/hDPP4 transgenic mice and type I interferon receptor (IFNAR)-KO mice [84]. The human anti-RBD antibody 3B11-N is another promising candidate that prophylactically reduces lung pathology in rhesus monkeys infected with MERS-CoV [85].

Targeting the S1-DPP4 interaction from the host side through the development of anti-DPP4 (CD26) antibodies is another possible therapeutic option. The anti-CD26 antibodies 2F9, 1F7 and YS110 target the MERS-CoV entry into cells *in vitro* [86]. The 2F9 epitope maps close to the binding site of ADA, a natural DPP4 binding protein and MERS-CoV antagonist, while the 1F7 and YS110 epitopes lie outside this region [55, 86].

Thus, targeting of the S1-DPP4 interaction by use of mAbs is a promising strategy for the clinical development of molecular therapeutics against MERS-CoV. Another molecular approach involves targeting of the S2-mediated MERS-CoV-host cell fusion element of the MERS-CoV infection cycle by use of antiviral peptides.

## Antiviral peptides: HR2 region of S protein

The role of HR1 and HR2 in viral fusion makes them potentially effective molecular therapeutic targets. This has been borne out by *in vitro* and *in vivo* results obtained using HR2 peptides (Table 1). HR1 peptides are ineffective antivirals as they aggregate in physiological solutions [87–89].

A peptide named HR2P, which spans residues 1251–1286 of HR2, effectively inhibits viral replication and S protein-mediated cell fusion *in vitro* [87]. A HR2P analogue named HR2P-M2 is an even more potent fusion blocker *in vitro,* and inhibits MERS CoV-expressing pseudovirus infection [90]. HR2P-M2 interacts with an HR1 peptide to effectively block 6HB bundle formation. *In vivo* HR2P-M2 intranasal administration to Ad5/hDPP4 transgenic mice protected them from MERS-CoV infection, as evidenced by the reduction of the lung viral titres by more than 1000-fold [90]. The addition of IFN-β along with HR2P-M2 enhanced the protective effect [90]. Thus, S2 HR2 peptides have potential as MERS-CoV intranasal antiviral treatments.

## Vaccine candidates

### S protein targeting

The S protein is also the focus of a number of candidate vaccines (Table 1) [75, 91–93]. A fusion product combining truncated RBD and the Fc portion of human IgG can bind human DPP4 and inhibit MERS-CoV infection in an *in vitro* cell culture model [91]. Importantly, this RBD–IgG fusion product can induce a humoral response in mice vaccinated by subcutaneous injection, hence blocking RBD–DPP4 binding and inhibiting MERS-CoV infection [91]. Further *in vivo* studies have indicated that intranasal administration to mice induces similar long-term IgG humoral responses to those achieved with subcutaneous injection, but superior cellular immune responses and local mucosal responses in lungs [92, 93]. This suggests that this type of construct is both potentially effective and readily deliverable by intranasal means. Use of an adjuvant, particularly MF59, significantly improves the humoral and T cell immunogenicity of the RBD s377-588–Fc IgG fusion construct in subcutaneously immunized mice [94]. The possibility of using the S1 RBD as a vaccine molecular target for a range of divergent MERS-CoV strains and escape mutants has also been explored recently [95]. The use of five recombinant RBDs with mutations observed in different MERS-CoV outbreaks or in camel strains induced potent neutralizing antibody responses against several MERS-CoV pseudoviruses [95].

However, while the RBD of the S1 subunit is a logical and promising target for MERS-CoV vaccine development, the epitope scope is relatively limited and full-length S protein may be a preferable option [75]. Technical difficulties in stably expressing abundant quantities of full-length S protein have presented a barrier. However, studies on delivery options, including the use of adjuvants and nanoparticles, may help in overcoming such issues. One study undertaken by Novavax (Gaithersburg, Maryland, USA) showed that the inoculation of mice by intramuscular injection with full-length S protein proprietary nanoparticles produced a relatively low neutralizing antibody response after 21 days [96]. However, the addition of the adjuvants Alum or Matrix M1 resulted in a robust and sustained anti-MERS-CoV neutralizing antibody response [96].

### Viral vectors

Other potential vaccination strategies include the use of live attenuated viruses, recombinant viruses or DNA plasmids expressing MERS-CoV genes. Various types of viral vectors are currently under development for use in potential MERS-CoV vaccines, including modified vaccinia virus Ankara (MVA), ad5- or ad41-type adenoviruses and measles viruses, all of which have good safety profiles (Table 1) [97–100]. Vaccination of mice by subcutaneous or intraperitoneal injection with MVA expressing full-length S protein induces robust and sustained MERS-CoV-specific neutralizing antibody and cytotoxic T lymphocyte responses, including in mice expressing human DPP4 [98]. These viruses are expected to enter clinical trials as a proposed prophylactic MERS-CoV vaccine. Likewise, intramuscular injection of ad5 or ad41 expressing full-length S protein induces both antigen-specific T cell and neutralizing antibody responses in mice [99]. Finally, intraperitoneal injection of measles virus expressing either membrane-anchored, full-length S protein or soluble S protein lacking the TM domain induces robust MERS-CoV antigen-specific neutralizing antibody and cytotoxic T lymphocyte in interferon-α/β receptor (IFNAR)-deficient mice [100]. Recently, an MVA-based vaccine expressing S protein has been shown to induce mucosal immunity in MERS-CoV-infected dromedary camels, with a reduction in excreted virus and viral transcripts [101]. This has potential for veterinary use and the reduction of cross-species infection of humans by camels [101].

### DNA plasmids

GLS-5300 is a DNA-plasmid vaccine that encodes MERS-CoV S protein (Table 1) [102, 103]. It was co-developed by Inovio, GeneOne Life Science, Inc. and the Walter Reed Army Institute of Research, and has become the first potential MERS-CoV vaccine to enter human testing [102, 103]. A phase I clinical trial in healthy volunteers commenced in 2016 for the evaluation of its safety and ability to generate antibody and cellular immune responses over a 1-year period, using one of three dosages in a three-injection regimen [102]. The vaccine has already undergone pre-clinical trials in mice, camels and macaques [103]. It induced robust and antigen-specific cytotoxic T lymphocyte and neutralizing antibody responses, which effectively protected animals against viral infection [103].

GLS-5300 and other potential vaccine candidates provide an opportunity to develop a prophylactic MERS-CoV vaccine. However, the barriers to development of a prophylactic vaccine include the current relatively low MERS-CoV incidence in humans, as well as sourcing suitable small animal models [75, 97, 104]. These factors complicate the definition of a target population for mass prophylactic vaccination and pre-clinical demonstration of vaccine efficacy [104]. In this context, the monoclonal antibodies described above may be invaluable resources in an outbreak situation [76–85].

## Interferons: monotherapy and combination therapy

### *In vitro* and animal studies

While MERS-CoV-specific therapies are offering promising pre-clinical results, and GLS-5300 has entered clinical trials, there is as yet no specific evidence-based therapy or vaccine clinically available for MERS-CoV. As described in the MERS-CoV infection and replication section, MERS-CoV accessory protein products are IFN antagonists [62, 63]. Attenuation of the IFN response is an important MERS-CoV immune response circumvention mechanism [105]. The ORF4a in particular inhibits IFN-β production via the inhibition of interferon regulatory transcription factor (IRF)-3 and nuclear factor (NF)-κB actions, and thus IRF-3-activating small molecules, for example, may be potential therapeutic agents for restoring IFN responses [62, 63]. Toll-like receptor-3 (TLR-3) is also involved in the immune response of mice to SARS-CoV and MERS-CoV, recognizing viral molecular patterns and initiating the innate response that leads to IFN production (Fig. 2) [106]. Thus, TLR-3 agonists are another possible candidate for MERS-CoV-specific anti-viral agents [106].

Therapeutically, IFN itself is particularly useful prophylactically or during the early days of viral exposure, including for coronaviruses [105, 107]. *In vitro* and animal studies have confirmed the potential efficacy of IFNs in MERS-CoV therapy, in particular in combination with other therapeutic agents such as ribavirin and/or lopinavir. *In vitro*, MERS-CoV was substantially more susceptible to IFN-α than SARS-CoV [107]. While MERS-CoV in Vero or LLC-MK2 cells was sensitive to both IFN-α2b and ribavirin separately, relatively high concentrations were required to reduce viral replication [108]. However, combination therapy allowed the concentrations of each to be substantially reduced [108]. Combination therapy of IFN-α2b and ribavirin in macaques administered 8 hours after MERS-CoV infection reduced systemic and local viral effects, and reduced viral genome copy number and gene expression levels [109]. Bioinformatics data from microarray analysis recently showed that IFN-α2b and ribavirin treatment impacts on MERS-CoV gene expression in 10 different pathways, including genes involved in recognition of pathogens, immune responses and release of cytokines [110]. Both IFN-β1b and lopinavir treatment, alone or in combination, also protected marmosets from the adverse clinical, radiological and pathological effects of MERS-CoV infection [111].

### Clinical studies

Clinically, the use of IFN monotherapy, or IFN therapy in combination with ribavirin and/or lopinavir/ritonavir, has shown some promise (Table 1) [37–40]. However, the interpretation of clinical studies has been complicated by variability in factors such as the stage of infection at which therapy was administered. The available data are limited to case studies and retrospective cohort studies [22, 40]. In one case study on a patient who died in a Greek hospital, pegylated IFN along with ribavirin and lopinavir was administered as part of the treatment regime, but not until the thirteenth day of the illness [39]. By contrast, in another preliminary study on two patients, the first patient was treated with IFN-α2b and ribavirin within a day of admission prior to MERS-CoV diagnosis, but he was also being treated with antibiotics, steroids and non-invasive ventilation [37]. Patient 2, the wife of patient 1, was treated prophylactically after developing a low-grade fever and poorly defined lung infiltrates, but a diagnosis of MERS-CoV was not formally made [37]. Thus, while patient 1 survived and patient 2 had only a mild course of illness, it is difficult to draw any firm conclusions regarding the efficacy of the treatment. In another case study on a patient in Korea, administration of pegylated IFN-α2a along with ribavirin and lopinavir 4 days after hospital admission was deemed to have been effective in viral clearance and patient survival [38]. These case studies do not overall provide firm evidence for the efficacy or otherwise of IFN combination therapy for MERS-CoV.

In one case involving a series of five patients who were critically ill with MERS-CoV infection and on mechanical ventilation and corticosteroids, IFN-α2b and ribavirin was administered on average 19 days after admission [27]. All five patients died, but they may not have benefited, as they were treated late in their illness and were already critically ill [27]. The benefit of earlier treatment in less vulnerable patients was suggested in another series of six patients in which three who received IFN-α2b and ribavirin early in the illness survived, while three other patients who were older and had comorbid conditions received the combination treatment later and all died [112]. However, in another study in which 20 mechanically ventilated patients with severe MERS-CoV infection who received pegylated IFN-α2a and ribavirin early in treatment were compared to 24 patients who did not receive the combination therapy, the 14-day survival rate was significantly higher in the treatment group, but the 28-day survival rate was equivalently low in the two groups [113]. In another retrospective analysis of results from a series of 32 patients who received either IFN-α2a or IFN-β1a in combination with ribavirin, no significant difference in outcome between the two types of IFN was shown, and there was no survival benefit due to use of either IFN [29]. However, most of the patients in this study were aged more than 50 years and some had comorbid conditions, including end-stage renal disease [29]. Thus the retrospective studies that have been carried out are heterogeneous in terms of type of patient, stage of disease and type of IFN used, including whether or not it was pegylated or short-acting. There is an urgent need for well-controlled clinical trials for IFN combination therapy in MERS-CoV, preferably early in the illness, as IFNs are routinely available agents whose safety and efficacy is established for other viral illnesses and whose use has a sound molecular basis for MERS-CoV treatment.

## Protease inhibitors

### S protein proteases

Another type of therapy with a logical molecular basis for MERS-CoV treatment is the targeting of proteases, both host and viral (Table 1; Fig. 2) [19, 23, 53, 59, 60, 114–117]. Camostat, an inhibitor of TMPRSS2, is a potential therapeutic agent for coronaviruses such as SARS-CoV and MERS-CoV [59]. In a pathogenic mouse model of SARS-CoV infection, viral spread and pathogenesis was effectively blocked by camostat, and it is likely that it would have a similar impact on MERS-CoV [59]. As camostat is already in clinical use for the treatment of chronic pancreatitis, it represents a potentially safe and effective therapeutic option. Recently, another TMPRSS2 inhibitor, nafamostat, was identified in a split protein-based cell–cell fusion assay as a potent inhibitor of MERS-CoV S protein-mediated host–viral membrane fusion *in vitro* [118]. Nafamostat is already clinically approved for use by the US Food and Drug Administration (FDA) and is used as an anticoagulant [118]. The cathepsin L inhibitor teicoplanin, a glycopeptide antibiotic, was recently shown, via high throughput screening of FDA-approved drugs, to block entry of MERS-CoV, SARS-CoV and Ebola pseudoviruses into the cytoplasm [119]. Teicoplanin is currently used clinically as an antibiotic in both prophylaxis and the treatment of serious Gram-positive bacterial infections. It also has derivatives, including dalbavancin, oritavancin and telavancin, all of which also block viral entry.

### Viral proteases

#### PL(pro) inhibitors

The viral proteases, Mpro (3CLpro) and PL(pro), also represent potential molecular therapeutic targets [57, 60]. As well as its role in viral maturation, the MERS-CoV PL(pro) causes deubiquitination of IFN regulatory factor 3 (IRF-3), and hence suppression of IFN β production, which contributes to viral suppression of the innate immune response (Fig. 2) [120, 121]. The X-ray 3D crystal structure of MERS-CoV PL(pro) is similar to that of SARS-CoV, and includes ubiquitin-like and catalytic core domains [120]. Thus the SARS-CoV PL(pro) inhibitors, 6-mercaptopurine (6MP) and 6-thioguanine (6TG), can inhibit MERS CoV protease activity *in vitro* [61]. However, the MERS-CoV PL^pro^ crystal structure also has unique aspects, including the oxyanion hole, and S3 and S5 subsites, which may be viable molecular targets for antivirals specifically designed against MERS-CoV [120]. A commercial compound termed compound 4 (commercial code F2124–0890,Life Chemicals) has been identified as an inhibitor of MERS-CoV and SARS-CoV PLpro activity [122, 123]. The critical binding interactions and mode of inhibition differ between the two viral proteases, with the compound acting as a competitive inhibitor against MERS-CoV PL(pro), but an allosteric inhibitor of SARS-CoV PL[pro) [122]. However, F2124–0890 may lose potency in physiological reducing environments [123].

#### Mpro inhibitors: lopinavir/ritonavir

Lopinavir is a protease inhibitor with activity against the SARS-CoV main protease M^pro^ [124]. In a screen of a library of 348 FDA-approved drugs to identify anti-MERS-CoV activity in cell culture, lopinavir emerged as one of four compounds that inhibited viral activity in a low micromolar range [125]. However, the clinical efficacy of lopinavir in MERS-CoV treatment has not yet been fully established. As mentioned above, it has usually been used clinically in combination with IFN and data are only available from case studies and series. However, notably, lopinavir–ritonavir treatment resulted in better clinical, radiological and pathological outcomes and reduced mortality in marmosets infected with MERS-CoV [36]. Lopinavir has also been identified in a position paper from PHE and ISARIC–WHO as a potential MERS-CoV therapy whose benefits are likely to exceed its risks [47].

## Conclusions

Thus far, MERS-CoV has not been considered to have pandemic potential. Most cases have occurred in the Middle East, particularly in KSA. Outbreaks have been primarily linked to healthcare institutions, and shortcomings in infection control and prevention procedures [6–14]. However, potential viral mutations could facilitate expanded viral host range and enhance cross-species and human–human transmission [20, 58, 114]. The outbreak in Korea resulted in MERS-CoV emergence in second- and third-generation contacts, highlighting the potential for mutational changes that could increase the likelihood of human–human transmission [14, 18]. MERS-CoV also exacts a high mortality rate, mainly due to the development of ARDS [15–17]. These factors emphasize the importance of developing targeted therapies and/or vaccines. The most promising advances in the development of specific molecular MERS-CoV therapies relate to targeting of the viral S protein by means of anti-S1monoclonal antibodies, HR-targeted antiviral peptides and viruses or plasmids bearing S protein as potential vaccine candidates [52, 55, 58, 88–105]. The use of IFNs, usually in combination with other therapies such as ribavirin or lopinavir, also has a logical molecular basis given that IFN antagonism is an important mechanism by which the virus circumvents the innate immune system [62, 63, 105, 107]. Targeting of host and viral proteases is also a sound molecular approach, as host proteases are important in viral–host membrane fusion, while viral proteases are key to viral maturation and are also involved in targeting IFNs [23, 53, 59–61, 114–117].

The therapies currently used for MERS-CoV have mainly been extrapolated from those used for SARS-CoV treatment, regardless of the important differences in receptor usage and cellular tropism between the viruses [20–26]. None of these therapies have been subject to well-controlled trials, and in some cases the risks are likely to outweigh any poorly defined benefits [19, 22, 27–42]. In general, the clinical research response to MERS-CoV may have been too slow [126]. Thus, while there are many promising lines of research in terms of specific molecular targeting of MERS-CoV, no potential therapies have yet been subject to well-designed clinical trials, and none have been approved for clinical use, apart from the GLS-5300 DNA-plasmid vaccine [102, 105]. Continuing outbreaks of MERS-CoV, with possible increases in human–human transmission, are likely to galvanize the research community to push ahead with the design and performance of clinical trials for some of the available monoclonal antibodies and/or antiviral peptides for use in outbreak situations.

There are various challenges inherent in the development of specific MERS-CoV therapies. These include the difficulty of identifying a target population for potential prophylactic vaccines, limited small animal model availability and dependence on transgenic mouse models, and the current relatively low incidence of infection, which complicates the performance of adequate clinical trials [75, 96, 97, 104]. For example, one currently ongoing trial on convalescent plasma therapy has been affected by logistical and technical issues, including insufficient available donors and difficulty in collecting convalescent plasma containing sufficient MERS-CoV antibody levels [71, 72]. Thus, while numerous monoclonal antibodies have been raised with anti-MERS activity, in particular against the S protein [76–85], and promising antiviral HR2 peptides have been synthesized [87, 90], the available data are thus far limited to *in vitro* and animal studies.

Despite these issues, there is cause for optimism, given the many candidate antibody and peptide therapies. There is also some promising *in vitro* and animal model evidence suggesting that use of IFNs, which are well-established therapies in other viral illnesses, may be of benefit if used sufficiently early in MERS-CoV treatment, or as a prophylactic, especially in combination with other therapies, including ribavirin or lopinavir–ritonavir [108–111]. Likewise, other drugs that are currently in clinical use for other conditions have been shown to be potentially useful for MERS-CoV treatment, including camostat and nafamostat; teicoplanin and its derivatives dalbavancin, oritavancin and telavancin; and the SARS-CoV PL(pro) inhibitors, 6-mercaptopurine (6MP) and 6-thioguanine (6TG) [59, 61, 118–121]. These drugs have already been shown to be safe and well-tolerated by humans. Repurposing of existing drugs may therefore prove to be the most viable option in MERS-CoV therapy. For example, 1 screen of 290 approved drugs uncovered 27 candidates with *in vitro* activity against both MERS-CoV and SARS-CoV, including oestrogen receptor inhibitors and dopamine receptor inhibitors [127]. Thus, there are many options available on a molecular level for the development of new MERS-CoV-specific therapies, as well as the adoption of drugs that are currently in use for other purposes, which should assist in more effective and reliable prevention and treatment of this virus.
